# Infection respiratoire aigüe et statut nutritionnel chez les enfants de 0-5 ans: cas des cliniques universitaires de Lubumbashi, République Démocratique du Congo

**DOI:** 10.11604/pamj.2014.19.393.5248

**Published:** 2014-12-18

**Authors:** Léon Kabamba Ngombe, Mbombo-Ditunga, Nduwa Kameya, Aimé Abasiko Malingo, Nathalie Kaj Kayomb, Jean Ngolomba ea Ngolomba, David Kakez Nday, Luboya Numbi

**Affiliations:** 1 Université de Lubumbashi, Faculté de Médecine, Département de Santé Publique, DEA 9ème Promotion, RD du Congo; 2 Université de Kamina, Faculté de Médecine, Département de Santé Publique, Unité de Toxicologie, République Démocratique du Congo

**Keywords:** Infections respiratoires aiguës, enfants, statut nutritionnel, ARI, children, nutritional status

## Abstract

Les auteurs rapportent les données d'une étude rétrospective de 153 dossiers d'enfants hospitalisés dans le service de pédiatrie des Cliniques Universitaires de Lubumbashi/RD Congo pour IRA. En ce qui nous concerne, les IRA chez les enfants de moins de 5 ans représentent 26,11% de l'effectif, dont 17,75% âgés de moins d'un an. Le sexe masculin est légèrement prédominant (85 contre 68) et près de 70% des enfants ont un statut nutritionnel précaire. La répartition mensuelle connaît des pics en mars et octobre. Les diagnostiques notifiés sont: rhinite(16,3%),Amygdalite(5,9%),Otite Moyenne Aigue(0,7%),Laryngite (3,3%), Rhinopharyngite (39,2%), Pharyngite (6,5%), Bronchite (7,2%), Bronchopneumonie (5,9%), Pneumonie(2,6%) et Bronchiolite (12,4%).Tous les cas ont bien évolués sous traitement. Le but de ce travail est de déterminer la fréquence des IRA et le statut nutritionnel des enfants ayant été admis dans le service des pédiatries aux C.U.L.

## Introduction

Les infections respiratoires aiguës (IRA) sont une des causes de décès les plus importantes chez les jeunes enfants dans les pays en développement. Selon les plus récentes estimations de l'OMS, dans le monde, les IRA sont à elles seules responsables de 18,1% des décès chez l'enfant [1]. Par nécessité dictatique, les pathologies de la sphère ORL notamment les rhino pharyngites, amygdalites, pharyngites font partie des IRAH et que l'atteinte infectieuse du parenchyme pulmonaire, des bronches et de la trachée vont parti des IRAB. Ainsi, les infections respiratoires basses, surtout les pneumonies sont les principales causes de morbidité et de mortalité chez les enfants en bas âge dans les pays en voie de développement [2]. On estime qu'environ 25% des décès avant l’âge de 5 ans sont imputables aux IRA dans ces pays [3]. Les IRA sont responsables de 1/4 à 1/3 des décès frappant les nourrissons de bas âge [3]. Certains facteurs favorisent la survenue des IRA chez les enfants, c'est le cas de la pauvreté, l'absence de vaccination et d'hygiène, la promiscuité [4]. En RD Congo, nous avons une prévalence de 6% de la pneumonie chez les enfants de 0-5 ans [5] et EDS-RDCII 2013-2014 donne une prévalence de 7% des IRA chez les enfants de moins de 5ans. Nous avons voulu par ce travail déterminer la fréquence des IRA et le statut nutritionnel des enfants ayant été admis dans le service des pédiatries aux cliniques universitaires de Lubumbashi.

## Méthodes

Notre étude rétrospective a porté sur les enfants de 0-5 ans ayant consulté le service de pédiatrie des cliniques universitaire de Lubumbashi durant toute l'année 2013. Sur un total de 586 dossiers, seul 153 dossiers ont été retenu. Pour chaque dossier d'enfants, les éléments suivant ont été analysés: le poids, l’âge, le sexe, l′examen clinique des patients,le diagnostic posé et les symptômes en cause de la consultation tels que: fièvre, toux, écoulement nasal, problème d′oreille ont permis de faire la sélection des patients à partir de leur dossiers médicaux respectifs. Le statut nutritionnel de chaque enfant a été calculé à l'aide du logiciel ANTHRO qui a permis de classer l'enfant comme suit: poids normal par rapport à l’âge avec Z-Score de 0 Ecart type; enfants avec malnutrition globale (Emaciation et retard de croissance) légère avec Z-score <-1 à -2 Ecart type; modérée avec Z-Score <-2 à -3; Sévère avec Z-Score < -3. Cependant, l'analyse des données a été faite avec les logiciels Epi Info 7 et Excel 2010.

## Résultats

**Figure 1:**
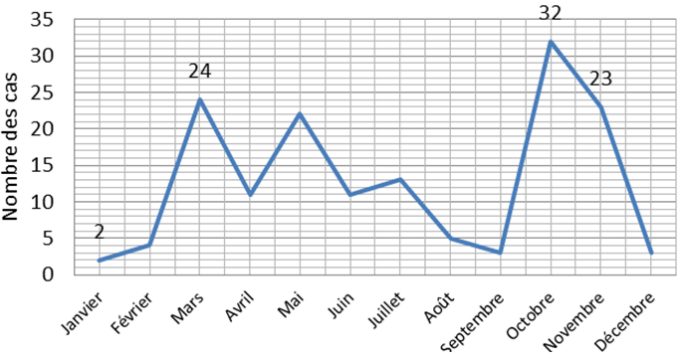
Répartition mensuelle de cas des IRA hospitalisés

**Figure 2:**
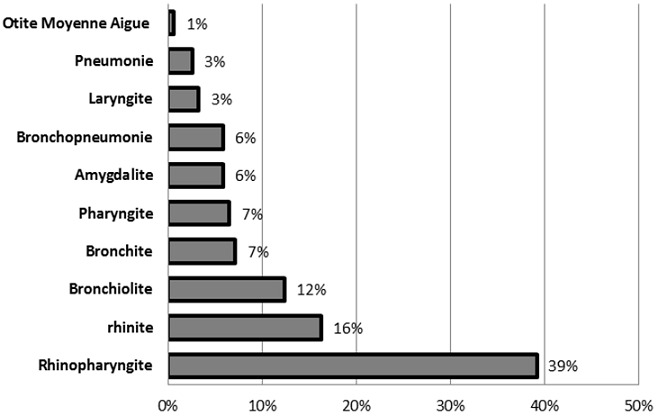
Répartition de cas des IRA selon le diagnostic clinique

Sur les 153 dossiers d'enfants hospitalisés dans le service de pédiatrie des Cliniques Universitaires de Lubumbashi pour IRA, les enfants de moins de 5 ans représentent 26,11% de l'effectif, dont 17,75% âgés de moins d'un an (Tableau 1). Le sexe masculin est légèrement prédominant soit un sex ratio de 1.25 (Tableau 2). On a trouvé que les IRAH était fréquente chez les enfants soit 71,9% (Tableau 3); la majorité des enfants (70%) avait un statut nutritionnel précaire (Tableau 4). En ce qui concerne La répartition mensuelle, on a noté des pics en mars et octobre (Figure 1). Dans notre serie, les diagnostiques notifiés incluaient: rhinite(16,3%),Amygdalite(5,9%),Otite Moyenne Aigue(0,7%),Laryngite (3,3%), Rhinopharyngite (39,2%), Pharyngite (6,5%), Bronchite (7,2%), Bronchopneumonie (5,9%), Pneumonie(2,6%) et Bronchiolite (12,4%) (Figure 2). L’évolution de tous les cas était bonne sous traitement faits des antibiotiques.

**Tableau 1:** Fréquence de cas des IRA hospitalisés aux C.U.L

Type d'hospitalisation	Effectif	%
Hospitalisation nourrisson et enfants	586	100%
IRA du nourrisson et enfants étudiés	153	26,1%
IRA Basse du nourrisson et enfants étudiés	43	7,3%
IRA Haute du nourrisson et enfants étudiés	110	18,8%

Ce tableau révèle que la proportion élevée est attribuée aux IRAH (18,8%)

**Tableau 2:** Répartition de cas des IRA en fonction de la tranche d’âge et du sexe

Tranches d’âges	M (%)	F (%)	Total	%
0 – 6	43(57%)	32(42, 6%)	75	49,0%
6 – 12	20(54%)	17(45, 9%)	37	24,2%
12 – 24	13(49, 9%)	13(49, 9%)	26	17,0%
24 – 59	9(60%)	6(40%)	15	9,8%
Total	85	68	153	100,0%

Ce tableau montre que la grande portion de cas des IRA concerne la tranche d’âge de 0-6 mois et que le sexe masculin est prédominant

**Tableau 3:** Répartition des IRA en fonction de la tranche d’âge

Ages (mois)	IRA Haute		IRA Basse		Total	
0 - 6	53	(34,6%)	22	(14,4%)	75	(49,0%)
6 - 12	24	(15,7%)	13	(8,5%)	37	(24,2%)
12 - 24	20	(13,1%)	6	(3,9%)	26	(17,0%)
24 - 59	13	(8,5%)	2	(1,3%)	15	(9,8%)
Total	110	(71,9%)	43	(28,1%)	153	(100,0%)

Ce tableau révèle que la tranche d’âge de 2-5 ans est la moins touchée par les IRA

**Tableau 4:** Répartition de cas des IRA en fonction de l’état nutritionnel

	Statut nutritionnel			
Diagnostique	Malnutrition	Pas de malnutrition	Total	%
IRA Haute	43	67	110	72%
IRA Basse	20	23	43	28%
Total	63	90	153	100%
%	41,2%	58,8%	100%	

Ce tableau révèle que le cas de malnutrition élevé ont été retrouve chez les patients présentant les IRAH (68%)

## Discussion

La fréquence hospitalière des IRA dans notre étude représente 26,1%, et est reparti de la manière suivante: IRA haute 18,8% et IRA basse 7,3%. La fréquence trouvée dans notre étude concernant les IRA basse est élevée par rapport à celle trouvée au Togo par B.BAKONDE [6]. En ce qui concerne les IRA haute chez nous, le premier rang est occupé par la rhinopharyngite avec 39%, rhinites 16%, pharyngite 7%, laryngite 3%,otite 1%. Nos fréquences en ce qui concernent les IRA haute sont inférieures à celles trouvées au Madagascar par H. RAOBIJAONA [7]. Cette fréquence est biaisée à cause de plusieurs facteurs notamment: le coût élevé de consultation décourages les parents à faible revenu qui préfèrent consulter à moindre frais ailleurs, le biais de recrutement par le fait que les cliniques est une structure de référence secondaire. Notre taux hospitalier de 26,1% se rapproche des taux trouvés par certains auteurs africains qui variaient entre 26,7-32,7% [8, 9]. Nos résultats sur la répartition des IRA en fonction de l’âge montre que la tranche d’âge de 0 à 1 ans semble la plus représentée dans notre étude. En rapport avec les IRA basse, cette tranche représente à elle seule près de 81,4% (35 cas/43) alors que la tranche de 2- 5ans est moins représentée avec 4,7%(2 cas/43). Ce résultat est supérieur à celui trouvé par B.BAKONDE au Togo par [6]. Pour les IRA haute, la tranche de 0-1 ans représente 70% (77 cas/110);et ce résultat est supérieur à celui trouvé par H. RAOBIJAONA [7] et RAVAOARINORO [10] au Madagascar. Nos données concernant l’âge confirme le fait que les IRA constituent un problème réel de sante publique parmi les enfants de moins de 5 ans déjà évoqué par plusieurs auteurs et par l'OMS [11–14].

Dans notre étude, le sexe masculin est légèrement plus représenté que le sexe féminin avec un sex-ratio à 1,25 qui n'est pas diffèrent de celui trouvé de par B.BAKONDE [6]. Par ailleurs, certains auteurs évoquent cette notion d'atteinte préférentielle du sexe masculin en cas des IRA [15–19]. La variation saisonnière joue un rôle important dans la survenue des IRA. Dans notre étude nous avons observé des pics au mois de Mars et Octobre qui correspondent respectivement au début de la saison sèche et début saison des pluies. Ces variations saisonnières confirment celles trouvées par B.BAKONDE [6], MINGA L.A. [20] en 1990 et KATCHALLA [21] au Togo. Concernant le statut nutritionnel, nous avons eu 41,2% de cas des IRA avec malnutrition aux Cliniques Universitaires de Lubumbashi. Ce chiffre est supérieur à celui trouvé par B.BAKONDE [6] et inférieur à celui trouvé par GUEDEHOUSSOU [18] au CHU Tokin où tous les indigents consultaient à la différence de CUL qui est consulté par une certaine classe sociale.

## Conclusion

La prévalence hospitalière trouvée dans cette étude montre que les IRA constituent un problème majeur de santé publique dans notre milieu. En effet, l’état nutritionnel précaire prédispose les enfants aux IRA. Il s'avère important de renforcer la prise en charge de l'enfant de manière intégrée dans les pays en voie de développement.
